# Effects of dietary fermented sweet potato residue on growth performance and cecal microbiota in sichuan white geese during the finisher period (28 to 70 days of age)

**DOI:** 10.1016/j.psj.2026.106437

**Published:** 2026-01-12

**Authors:** Jiayi Xu, Chao Wang, Guoan Yin, Shuai Zhao, Xin Liu, Weiguo Cui

**Affiliations:** a College of Animal Science and Veterinary Medicine, Heilongjiang Bayi Agricultural University,163319, Daqing, Heilongjiang, China; b Chongqing Academy of Animal Science, 402406, Chongqing Rongchang, China

**Keywords:** Fermented sweet potato residue, Sichuan white goose, Growth performance, Meat quality, Gut microbiota

## Abstract

This study aimed to evaluate the potential of fermented sweet potato residue (FSPR) as a partial substitute for corn in goose diets, determine its optimal inclusion level, and assess its effects on intestinal morphology, immune status, and cecal microbiota. A 42-day feeding trial was conducted with 144 twenty-eight-day-old male Sichuan white geese randomly assigned to one of four dietary treatments: a corn-soybean meal basal diet (CON) and three test diets replacing corn with 5%, 8%, or 12% of the corn with FSPR on a dry matter basis. Growth performance, carcass traits, meat quality, serum biochemistry, intestinal morphology, and cecal microbiota were comprehensively assessed. Dietary FSPR inclusion induced a significant, dose-dependent reduction in average daily feed intake (*P* < 0.001). However, feed conversion ratio was linearly improved (*P* < 0.001), which compensated for the reduced intake and sustained growth performance. Despite reduced intake, the 8% FSPR group achieved the highest final body weight, and the feed conversion ratio was significantly improved in the 8% and 12% FSPR groups (*P* < 0.001). Additionally, FSPR improved meat quality by maintaining higher postmortem thigh muscle pH. Systemically, supplementation enhanced immunoglobulins (IgM, IgG) and anti-inflammatory IL-10 without inducing pro-inflammatory markers. Ileal morphology was optimized, evidenced by a significantly increased villus height-to-crypt depth ratio (*P* < 0.001). Cecal microbiota analysis revealed that FSPR enriched beneficial taxa (e.g., *Phocaeicola, Lachnospiraceae*), enhanced microbial diversity, and upregulated metabolic pathways for carbohydrate and amino acid biosynthesis. In conclusion, FSPR effectively replaces corn in goose diets, enhancing feed efficiency, meat quality, and immunity, likely associated with through gut microbiota modulation, with 8% identified as the optimal inclusion level.

## Introduction

The poultry industry's reliance on corn and soybean meal is challenged by price volatility, necessitating the exploration of cost-effective and sustainable alternative feed resources (Alshelmani et al., 2021). Sweet potato residue **(SPR)**, a major by-product of starch processing, represents a promising candidate due to its substantial annual output and low cost; approximately 4.5–5.0 tons of fresh SPR are generated per ton of starch processed (Zou et al., 2024). However, the direct dietary incorporation of SPR is limited by its inherent nutritional deficiencies. For instance, unprocessed SPR typically contains high dietary fiber content and low crude protein levels (Ju et al., 2017). These characteristics constrain utilization in monogastric animal feeds. Solid-state fermentation using microbial consortia (e.g., *Aspergillus niger* and *Saccharomyces cerevisiae*) transforms SPR into fermented sweet potato residue **(FSPR)**, thereby enhancing its nutritional value through increased crude protein and reduced fiber content (Yao et al., 2024).

While the general benefits of using fermented agro-byproducts in animal diets are recognized, systematic research to establish the efficacy and optimal inclusion levels of specifically FSPR in geese is currently lacking (Sun et al., 2023; Lian et al., 2024). Geese, as herbivorous poultry, possess a distinctive physiological advantage for utilizing fibrous feeds, owing to their robust gizzard and highly developed cecal fermentation system, which enables them to efficiently extract energy from dietary fiber (Fang et al., 2023) The Sichuan white goose, a dominant indigenous breed in China, is particularly noted for its rapid growth and strong tolerance of coarse fodder (Chen et al., 2024). This physiological adaptation suggests that geese may utilize FSPR more effectively than other species. Moreover, while the immunomodulatory and gut-health-promoting effects of fermented feed metabolites are well-documented in other specie (Wang et al., 2026), it remains unknown whether FSPR elicits similar protective mechanisms in geese.

This study was therefore conducted to determine the optimal dietary inclusion level of FSPR for Sichuan white geese, with the hypothesis that optimal inclusion would enhance growth performance and overall health by improving feed efficiency, modulating the cecal microbiota, and enhancing immune status. This study comprehensively evaluated the effects of FSPR on growth performance, carcass traits, meat quality, serum biochemistry, intestinal morphology, and cecal microbiota.

## Materials and methods

### Ethics statement

All animal procedures in this study were approved by the Animal Ethics Committee of Heilongjiang Bayi Agricultural University (Approval No. DWKJXY2025061) and were conducted in accordance with the Guide for the Care and Use of Agricultural Animals in Research and Teaching (4th edition, 2020).

### Preparation of FSPR

SPR was obtained from Chongqing Yongchuan Weiyang Sweet Potato Plantation Co., Ltd. (Yongchuan District, Chongqing, China). Solid-state fermentation was performed by inoculating the substrate (adjusted to a moisture content of 65% and pH 6.0 with sterile water) with a microbial consortium. This consortium consisted of *Bacillus velezensis* (CGMCC NO.4669), *Aspergillus versicolor* (ATCC 11730, selected for its documented cellulolytic activity) (Jiang et al., 2024), *Lactobacillus plantarum* (DSM 20174), and *Saccharomyces cerevisiae* (ATCC 26603) at a volumetric ratio of 3:3:3:1. Each strain was suspended in sterile water (autoclaved at 121°C for 15 min) to a concentration of ≥1.0 × 10⁷ CFU/mL; the final total inoculum volume was 7% (vol/wt) of the dry matter. Fermentation was conducted under anaerobic conditions in a walk-in environmental chamber (maintained at 28°C and 50% relative humidity) for 4 days. The substrate was sealed in fermentation vessels to prevent oxygen and maintain an anaerobic environment throughout the process.

The FSPR was incorporated into the diets in its fresh, wet state (pH 4.4, moisture content ∼60% - 65%) without prior drying or grinding. Four experimental diets were formulated by substituting corn with FSPR at inclusion levels of 0% (CON), 5%, 8%, and 12% on a dry matter (DM) basis. For feed preparation, the calculated amount of fresh FSPR was homogeneously mixed with the dry ingredients, and the resulting mixture was processed into pellets using a feed pellet mill. To prevent spoilage, fresh batches of pelleted diets were prepared weekly and stored at 4°C. Although the inclusion of wet FSPR increased the moisture content of the final pellets, the acidic environment (pH 4.4) of the FSPR acted as a natural preservative, effectively inhibiting fungal proliferation. Additionally, the feed was visually inspected daily to confirm the absence of mold growth throughout the experimental period.

Samples of SPR and FSPR were dried and analyzed for dry matter (method 930.15), crude protein (method 984.13), ether extract (method 920.39), crude fiber (method 978.10), and ash (method 942.05) according to Latimer (2023a). Nitrogen-free extract (NFE) was calculated by difference. The amino acid profiles were determined using an automatic amino acid analyzer following acid hydrolysis (Latimer, 2023). The metabolizable energy (ME) values for SPR and FSPR were calculated using the equation by Carpenter and Clegg (1956):

ME(kcal/kg)=53+38×(CP+2.25×EE+1.1×NFE)

The nutrient profile of corn was obtained from the Chinese Feed Database (2020). The nutrient composition of corn, SPR, and FSPR is summarized in Table 1.

**Table 1:** Nutrient composition of Corn, SPR, and FSPR.

Item	Corn^1^	SPR	FSPR
DM, %	86.00	70.68	71.14
CP, %	8.00	3.27	4.36
Ash, %	1.20	2.68	2.39
EE, %	3.60	0.44	0.48
CF, %	2.30	8.58	6.76
ME, MJ/kg^2^	13.47	10.64	11.08
Lysine, %	0.24	0.15	0.21
Methionine, %	0.17	0.05	0.06
Threonine, %	0.29	0.12	0.19
Cystine, %	0.17	0.04	0.08
Tryptophan, %	0.06	0.03	0.05
Arginine, %	0.37	0.09	0.16

SPR: sweet potato residue; FSPR: fermented sweet potato residue; DM, dry matter; CP, crude protein; EE, ether extract; CF, crude fiber; ME, metabolizable energy; NFE: nitrogen-free extract. All data are presented on an as-fed basis.

1 Values for Corn are reference values from the Chinese Feed Database (2020)

2 The ME of SPR and FSPR were calculated based on the following equation: ME (kcal/kg) = 53 + 38 × (CP + 2.25 × EE + 1.1 × NFE) (Carpenter and Clegg, 1956), and then converted to MJ/kg (1 kcal = 4.184 kJ).

### Experimental design, diets, and animal management

A 42-day feeding trial was conducted with 144 healthy 28-day-old male Sichuan white geese of similar initial body weight. Birds were randomly assigned to one of four dietary treatments in a completely randomized design (CRD). Each treatment consisted of 6 replicates (pens), with 6 geese per pen. The experimental diets included a corn-soybean meal basal diet (control, CON). All diets were formulated to be isonitrogenous (approximately 17.2% CP) by adjusting the inclusion levels of soybean meal and soybean oil. In these test diets, corn was partially substituted with 5%, 8%, or 12% FSPR on a dry matter basis. Although the calculated Metabolizable Energy (ME) decreased slightly with increasing FSPR levels (Table 2), this formulation strategy was intentionally adopted to maximize the inclusion of fermentation metabolites and evaluate their potential to compensate for the energy deficit through enhanced gut function and feed efficiency. This gradient was designed to systematically evaluate the biological effects of FSPR, covering a low level (5%) to assess its basic efficacy and safety, a medium level (8%) to explore its optimal benefits, and a high level (12%) to determine its tolerance limit in geese. The composition and nutrient levels of the experimental diets are presented in Table 2. The geese were reared in small pens with raised wire-mesh floors positioned 60 - 70 cm above the ground, at a stocking density of 3 birds/m². All birds had ad libitum access to feed and water under natural lighting conditions. Husbandry management and environmental conditions were consistent across all groups, meeting the hygienic requirements for meat goose production. The trial was conducted at the Poultry Research Base (Rongcheng District, Chongqing, China).

**Table 2:** Ingredients and chemical composition of experimental diets (as-fed basis).

Ingredients, %	CON	5% FSPR	8% FSPR	12% FSPR
Corn	67.00	63.50	61.00	58.00
FSPR	0	5.00	8.00	12.00
Soybean meal	20.00	21.50	22.00	22.50
Alfalfa meal	3.00	2.00	1.50	0
Wheat bran	5.00	5.50	6.00	6.50
Soybean oil	1.00	1.50	2.00	2.50
Choline chloride	0.10	0.10	0.10	0.10
NaCl	0.30	0.30	0.30	0.30
Dicalcium phosphate (CaHPO₄)	1.00	1.00	1.00	1.00
Limestone	0.85	0.85	0.85	0.85
L-Lysine-HCl	0.25	0.25	0.25	0.25
DL-Methionine	0.25	0.25	0.25	0.25
Premix^1^	1.00	1.00	1.00	1.00
Total	100.00	100.00	100.00	100.00
Calculated nutrient composition^2^				
ME, MJ/kg	11.80	11.70	11.60	11.50
Crude protein, %	17.20	17.20	17.20	17.20
Crude fiber, %	4.10	5.30	6.00	6.80
Calcium, %	0.85	0.85	0.85	0.85
Available phosphorus, %	0.45	0.45	0.45	0.45
Amino acids				
Lysine, %	0.94	0.98	0.99	1.00
Methionine, %	0.49	0.50	0.50	0.49
Cystine, %	0.27	0.28	0.28	0.28
Threonine, %	0.57	0.59	0.60	0.60
Tryptophan, %	0.19	0.20	0.20	0.20
Arginine, %	0.95	1.00	1.01	1.02

CON: control diet; FSPR: fermented sweet potato residue.

1 The premix provided the following per kg of diet: Cu (CuSO_4_ 5H_2_O) 8 mg; Fe (FeSO_4_ H_2_O) 96 mg; Zn (ZnSO_4_ H_2_O) 80 mg; Mn (MnSO_4_ H_2_O) 100 mg; Se (Na_2_SeO_3_) 0.3 mg; I (KI) 0.4 mg; vitamin A (retinyl acetate) 6000 IU; vitamin D_3_ (cholecalciferol) 1500 IU; vitamin E (dl-α-tocopheryl acetate) 10 IU; vitamin K_3_ 2.4 mg; vitamin B_1_ 1.5 mg; vitamin B_2_ 5 mg; vitamin B_6_ 3 mg; vitamin B_12_ 0.02 mg; pantothenic acid 10 mg; nicotinic acid 50 mg; folic acid 0.5 mg; biotin 0.15 mg.

2 Nutrient levels were calculated based on the analyzed value of FSPR and standard table values for other ingredients.

### Growth performance

Initial body weight (IBW) and final body weight (FBW) of birds were measured at the start (28 d of age) and end (70 d of age) of the 42-d trial, respectively. Average daily gain (ADG), average daily feed intake (ADFI), and feed conversion ratio (FCR) were calculated for each replicate pen over the entire experimental period. The pen was considered the experimental unit for all statistical analyses of growth performance.

### Sample collection and slaughter procedure

At the end of the 42-day feeding trial (70 days of age), one goose per pen with a body weight closest to the replicate average was identified, marked, and fasted for 12 h with free access to water. On the following morning (71 days of age), the selected geese were humanely slaughtered by exsanguination following electrical stunning, a procedure conducted in accordance with standard commercial practices and the principles of the Guide for the Care and Use of Agricultural Animals. Blood samples were collected from the brachial vein immediately before slaughter (of the selected geese). Carcass traits, including dressing percentage, semi-eviscerated yield, eviscerated yield, breast muscle yield, thigh muscle yield, and abdominal fat yield, were measured according to the Chinese Agricultural Industry Standard NY/T 823-2020. Specifically, the dressing percentage, semi-eviscerated yield, and eviscerated yield were calculated based on the pre-slaughter live body weight. The yields of breast muscle, thigh muscle, and abdominal fat were expressed as a percentage of the eviscerated carcass weight.

### Muscle quality

Samples from the left pectoralis major and thigh muscles were collected immediately post-slaughter for quality analysis. Meat color (CIE L, a*, b*) was measured using a portable colorimeter (Konica Minolta, CR-400, Osaka, Japan). The instrument was calibrated with a standard white calibration plate (Y = 93.5, x = 0.3162, y = 0.3328) before each measurement session. Color measurements were performed at two postmortem time points: 45 min and 24 h. For the 24 h measurement, muscle samples were stored at 4°C in sealed, light-proof plastic bags to minimize desiccation and surface discoloration. At each time point, three readings were recorded at evenly spaced locations on each muscle sample (avoiding areas of visible connective tissue and fat), and the mean value was used for subsequent statistical analysis.

The pH was recorded at 45 min postmortem and 24 h postmortem by using a portable pH meter (pH-STAR, Matthaus, Berlin, Germany). The pH meter was calibrated with standard buffer solutions (pH 4.01 and 7.01) at 25°C before use, and the electrode was inserted 1 cm deep into the center of the muscle sample for each reading (Latimer, 2023).

Drip loss, cooking loss, and shear force were determined as described by Cama-Moncunill et al. (2020), with modifications to adapt to goose muscle characteristics: the cooking temperature was adjusted from 75°C to 80°C because preliminary tests indicate that 80°C achieves complete protein denaturation while preventing excessive toughening, thus meeting the standard criteria for evaluating poultry tenderness.

### Serum biochemical indicators

Blood samples were allowed to clot at room temperature (25 ± 1°C) for 30 min. Subsequently, serum was separated by centrifugation at 3,000 × g for 10 min at 4°C. The obtained serum was aliquoted into sterile 1.5 mL microcentrifuge tubes and stored at -20°C until analysis. Serum biochemical parameters were analyzed using a veterinary biochemistry analyzer (Model BS-480 Vet, Mindray, Shenzhen, China) with commercial reagent kits. Kits for liver function and metabolic profiles were from the same manufacturer (Catalog No. BS-800M; Batch No. MR20240301). Kits for immune and cytokine indices were from Shanghai Zhuocai Biotechnology Co., Ltd. (Catalog No. ZC-202401; Batch No. ZC20240215).

### Intestinal histomorphology

Segments from three regions of the small intestine were collected immediately after slaughter: the duodenum (a 5-cm segment taken immediately distal to the pylorus), the jejunum (from the midpoint of the small intestine proper), and the ileum (a 5-cm segment taken immediately proximal to the ileocecal junction). Each segment was gently flushed with ice-cold phosphate-buffered saline (PBS; 0.01 M, pH 7.4) to remove residual digesta and then fixed in 4% (vol/vol) paraformaldehyde solution at 4°C for 24 h. Fixed tissues were processed through standard histological procedures, including dehydration through a graded ethanol series, embedding in paraffin, sectioning at 5-μm thickness, and staining with hematoxylin and eosin (H&E) (Al-Khalaifah et al., 2025).

Villus height (VH) and crypt depth (CD) were measured using a light microscope (Model BA400, Motic China Group Co., Ltd., Xiamen, China) coupled with an image analysis system (Motic Images Plus 2.0, Motic China Group Co., Ltd.). Villus height was defined as the distance from the villus tip to the crypt-villus junction. Crypt depth was measured from the base of the crypt to the crypt-villus junction. For each intestinal segment, five intact, well-oriented villus-crypt units were measured per goose, and the mean values for VH and CD were calculated. The villus height to crypt depth ratio (H/D) was then determined for each sample.

### Cecal microbiota analysis

Cecal digesta samples (approximately 1 g per replicate) were collected aseptically from each slaughtered goose, immediately flash-frozen in liquid nitrogen, and stored at -80°C until DNA extraction. Total genomic DNA was extracted using the TIANGEN DNA Stool Kit (DP304, Tiangen Biotech Co., Ltd., Beijing, China) according to the manufacturer's instructions. The concentration and purity of the extracted DNA were verified using a spectrophotometer (NanoDrop, Thermo Fisher Scientific, USA). The V3–V4 hypervariable region of the bacterial 16S ribosomal RNA (rRNA) gene was amplified by polymerase chain reaction (PCR) using the primers 341F and 806R. Amplicons were subjected to high-throughput sequencing on an Illumina NovaSeq 6000 platform (2 × 250 bp paired-end) by Chengdu Lilai Biotechnology Co., Ltd. (Chengdu, China).

Sequencing data were processed using QIIME 2 for quality filtering, amplicon sequence variant (ASV) clustering, and taxonomic assignment against the SILVA 138 database. Alpha diversity (within-sample diversity) and beta diversity (between-sample diversity) analyses, along with subsequent statistical calculations, were performed using Python-based algorithms.

### Statistical analysis

All data were processed and analyzed using Python 3.9 with scipy.stats (v1.10.1) and statsmodels (v0.14.0) libraries. The pen was considered the experimental unit for growth performance (ADG, ADFI, FCR), and the individual goose served as the experimental unit for carcass traits, meat quality, serum biochemistry, and intestinal morphology. Data were tested for normality of residuals (Shapiro-Wilk test) and homogeneity of variances (Levene's test) prior to analysis.

Data were analyzed using a one-way ANOVA. This method was applied to growth performance, carcass traits, meat quality, serum biochemistry, and intestinal morphology. Orthogonal polynomial contrasts were performed to evaluate dose-response relationships. These contrasts tested for linear and quadratic trends across increasing dietary FSPR levels. The inclusion rates were 0%, 5%, 8%, and 12%. A P value less than 0.05 was considered statistically significant.

For the cecal microbiota, beta diversity was compared by permutational multivariate analysis of variance (PERMANOVA) with 999 permutations based on Bray-Curtis dissimilarities. Differences in taxonomic relative abundance were assessed using the Kruskal-Wallis test, followed by Dunn's post-hoc test with Benjamini-Hochberg false discovery rate (FDR) correction. Data are presented as mean ± SEM. Differences were considered significant at *P* < 0.05.

## Results

### Growth performance

The effects of replacing corn with FSPR on the growth performance of geese are presented in Table 3. The highest FBW and ADG, which were significantly higher than those in the 5% FSPR group (P < 0.05), were found in the 8% FSPR group which were significantly higher than those in the 5% FSPR group (*P* < 0.05). In contrast, the dietary inclusion of FSPR resulted in a reduction in ADFI, exhibiting both significant linear (*P* < 0.001) and quadratic (*P* = 0.019) downward trends. Consequently, the lowest FCR was observed in the 8% and 12% FSPR groups, which were significantly lower than the CON and 5% FSPR groups (*P* < 0.05), showing a clear linear improvement with increasing FSPR levels (*P* < 0.001).

**Table 3:** Effects of dietary FSPR on the growth performance of geese from 28 to 70 days of age.

Items	CON	5%FSPR	8%FSPR	12%FSPR	ANOVA (P)	Linear (P)	Quadratic (P)
IBW, g	1091.69 ± 2.43	1095.47 ± 5.53	1094.62 ± 3.71	1098.83 ± 11.84	0.908	0.5005	0.9256
FBW, g	3354.83 ± 20.32^ab^	3290.28 ± 17.78^b^	3396.13 ± 23.27^a^	3327.61 ± 20.8^ab^	0.004	0.9893	0.9097
ADG, g	53.85 ± 0.50^ab^	52.29 ± 0.46^b^	54.83 ± 0.56^a^	53.76 ± 0.86^ab^	0.035	0.5717	0.5519
ADFI, g	202.21 ± 0.33^a^	197.7 ± 1.15^b^	192.47 ± 1.37^c^	183.74 ± 1.00^d^	<0.001	<0.001	0.0195
FCR	3.77 ± 0.04^a^	3.79 ± 0.04^a^	3.53 ± 0.05^b^	3.44 ± 0.05^b^	<0.001	<0.001	0.1315

Data are presented as mean ± SEM (n = 6).

^a-d^ Means within a row with no common superscript letters differ significantly (Tukey's HSD test, P < 0.05).

When the ANOVA P-value was < 0.05, orthogonal polynomial contrasts were tested for linear and quadratic trends. Significant trends (P < 0.05) are indicated in bold.

The feed conversion ratio (FCR) has been corrected to exclude the feed intake of deceased birds (total mortality: n=3).

Abbreviation: IBW, initial body weight; FBW, final body weight; ADG, average daily gain; ADFI, average daily feed intake; FCR, feed conversion ratio.

### Carcass traits

Carcass composition and meat yield of geese are presented in Table 4. Generally, dietary inclusion of FSPR did not significantly affect the carcass traits (*P* > 0.05). However, the highest numerical breast muscle yield was found in the 12% FSPR group. Notably, this parameter showed a tendency for a linear increase with higher dietary FSPR inclusion levels (*P* = 0.083). In contrast, the other measured traits, including dressing percentage, semi-eviscerated yield, eviscerated yield, thigh muscle yield, abdominal fat yield, lean meat yield, and skin and fat yield, were not significantly altered by the replacement of corn with FSPR (*P* > 0.05).

**Table 4:** Carcass traits and meat yield of geese fed diets containing different levels of FSPR.

Items	CON	5%FSPR	8%FSPR	12%FSPR	ANOVA (P)	Linear (P)	Quadratic (P)
Dressing percentage, %	83.78 ± 1.68	85.14 ± 1.13	84.05 ± 1.56	83.44 ± 0.43	0.849	0.867	0.454
Semi-eviscerated yield, %	75.59 ± 0.56	75.14 ± 0.99	78.11 ± 1.50	75.18 ± 0.51	0.179	0.657	0.415
Eviscerated yield, %	70.2 ± 0.51	69.81 ± 0.90	71.47 ± 1.87	70.66 ± 0.79	0.776	0.595	0.934
Breast muscle yield, %	6.49 ± 0.09	6.44 ± 0.14	6.56 ± 0.09	6.76 ± 0.07	0.198	0.083	0.184
Thigh muscle yield, %	18.24 ± 0.62	16.9 ± 0.33	17.82 ± 0.72	18.13 ± 0.66	0.546	0.943	0.249
Abdominal fat yield, %	3.22 ± 0.07	3.41 ± 0.13	3.32 ± 0.06	3.45 ± 0.09	0.383	0.155	0.783
Lean meat yield, %	23.37 ± 0.71	23.9 ± 0.62	22.78 ± 0.45	22.98 ± 0.54	0.585	0.448	0.757
Skin and fat yield, %	21.18 ± 0.37	20.4 ± 0.08	21.58 ± 0.45	21.31 ± 0.29	0.147	0.494	0.489

Data are presented as mean ± SEM (n = 6).

### Muscle quality

#### Water-holding capacity and shear force

As shown in Table 5, the water-holding capacity and shear force of breast and thigh muscles were not significantly affected by dietary treatments (*P* > 0.05). However, a quadratic trend was observed for cooking loss in thigh muscle (*P* = 0.072), with the lowest numerical values recorded in the 5% and 8% FSPR groups (27.40% and 27.55%, respectively). The shear force of the breast muscles across all groups ranged from 52.98 N to 55.22 N.

**Table 5:** Effects of dietary FSPR on water-holding capacity and shear force of goose meat.

Items	CON	5% FSPR	8% FSPR	12% FSPR	ANOVA (P)	Linear (P)	Quadratic (P)
**Breast muscle**							
Drip loss, %	11.04 ± 0.37	10.86 ± 0.32	11.46 ± 0.16	10.69 ± 0.24	0.289	0.690	0.366
Cooking loss, %	37.75 ± 1.30	37.24 ± 0.69	38.30 ± 0.77	36.35 ± 1.07	0.558	0.459	0.480
Shear force, N	55.22 ± 0.88	52.98 ± 1.35	53.97 ± 0.78	54.64 ± 1.03	0.474	0.768	0.160
**Thigh muscle**							
Cooking loss, %	29.94 ± 0.39	27.40 ± 0.95	27.55 ± 1.03	28.16 ± 0.76	0.144	0.144	0.072

Data are presented as mean ± SEM (n = 6).

#### Muscle pH

The postmortem pH values of breast and thigh muscles are presented in Table 6. The 24 h pH of thigh muscle was significantly affected by dietary treatment (*P* = 0.031), increasing linearly with higher FSPR inclusion (*P* = 0.048). The 12% FSPR group recorded the highest value, which was significantly greater than that of the 5% FSPR group. Significant quadratic responses were observed for the 45 min pH in both breast (*P* = 0.025) and thigh muscles (*P* = 0.046), as well as for the 24 h pH in breast muscle (*P* = 0.035). The 24 h pH of breast muscle also showed a tendency for a linear increase (*P* = 0.081). No other significant differences were detected among groups for the remaining pH measurements.

**Table 6:** Effects of dietary FSPR on postmortem pH of goose breast and thigh muscles.

Items	CON	5% FSPR	8% FSPR	12% FSPR	ANOVA (P)	Linear (P)	Quadratic (P)
**Breast muscle**							
pH _45 min_	5.55 ± 0.05	5.42 ± 0.04	5.47 ± 0.05	5.58 ± 0.07	0.294	0.488	0.025
pH _24 h_	5.42 ± 0.04	5.40 ± 0.01	5.41 ± 0.02	5.52 ± 0.05	0.146	0.081	0.035
**Thigh muscle**							
pH _45 min_	5.87 ± 0.09	5.70 ± 0.07	5.72 ± 0.09	5.92 ± 0.10	0.115	0.580	0.046
pH _24 h_	5.67 ± 0.07ab	5.55 ± 0.07b	5.75 ± 0.08ab	5.82 ± 0.06a	0.031	0.048	0.155

Data are presented as mean ± SEM (n = 6). Means within a row with different lowercase superscripts differ significantly (P < 0.05).

#### Meat color

The inclusion of FSPR altered several meat color parameters of goose breast and thigh muscles, primarily at the 24 h postmortem time point (Table 7). In the breast muscle, the L* value at 24 h was significantly affected (*P* = 0.002), with the lowest value observed in the 5% FSPR group, followed by the 12% FSPR group. For a*, no significant differences were found. However, b* varied significantly; at 45 min, the 5% FSPR group exhibited the lowest value, whereas at 24 h, the 8% FSPR group showed a significantly lower value compared to the CON group (*P* = 0.016).

**Table 7:** Effects of dietary FSPR on the meat color of goose breast and thigh muscles.

Items	Time	CON	5% FSPR	8% FSPR	12% FSPR	ANOVA (P)	Linear (P)	Quadratic (P)
**Breast muscle**								
L*	45 min	49.46 ± 4.28	47.58 ± 5.67	48.35 ± 4.43	48.27 ± 3.55	0.668	0.457	0.507
	24 h	51.77 ± 5.41^a^	46.97 ± 6.19^c^	53.37 ± 4.74^a^	50.65 ± 2.54^b^	0.002	0.942	0.957
a*	45 min	11.10 ± 2.47	11.67 ± 2.95	11.06 ± 2.03	11.75 ± 2.65	0.778	0.463	0.841
	24 h	13.06 ± 3.92	13.59 ± 3.55	11.4 ± 3.41	13.80 ± 3.00	0.164	0.960	0.895
b*	45 min	9.72 ± 1.17^a^	8.36 ± 1.45^c^	10.08 ± 1.30^a^	8.72 ± 1.62^b^	0.001	0.724	0.960
	24 h	10.68 ± 1.52^a^	9.93 ± 1.59^a^	8.88 ± 1.45^b^	9.66 ± 1.98^a^	0.016	0.284	0.457
**Thigh muscle**								
L*	45 min	39.95 ± 4.27	40.24 ± 3.84	38.88 ± 5.00	38.60 ± 6.27	0.701	0.176	0.493
	24 h	40.92 ± 5.14^a^	42.92 ± 6.80^a^	37.44 ± 5.65^b^	36.41 ± 3.80^c^	0.002	0.248	0.548
a*	45 min	10.67 ± 1.86	11.54 ± 2.22	11.03 ± 2.51	11.43 ± 1.95	0.604	0.354	0.678
	24 h	14.32 ± 2.62^a^	12.16 ± 1.57^b^	13.05 ± 1.17^a^	13.52 ± 1.51^a^	0.006	0.698	0.428
b*	45 min	9.26 ± 2.46	9.75 ± 1.55	8.78 ± 1.53	8.61 ± 1.52	0.235	0.333	0.595
	24 h	9.04 ± 2.43	8.60 ± 2.30	8.23 ± 2.18	7.66 ± 2.01	0.303	0.008	0.007

Data are presented as mean ± SEM (n = 6). Means within a row with different lowercase superscripts differ significantly (*P* < 0.05).

In the thigh muscle, the L value at 24 h* was highest in the 5% FSPR group and lowest in the 12% FSPR group (*P* = 0.002). Similarly, the a* at 24 h was significantly lowest in the 5% FSPR group compared to the CON, 8%, and 12% groups (*P* = 0.006). Notably, although no significant difference was detected in the mean values of b* for the thigh muscle at 24 h (*P* > 0.05), significant linear (*P* = 0.008) and quadratic (*P* = 0.007) downward trends were observed with increasing FSPR inclusion.

#### Serum biochemical

The serum biochemical profiles of geese are presented in Table 8. Dietary FSPR supplementation significantly affected the levels of immunoglobulins and anti-inflammatory cytokines. Specifically, the levels of IL-10, IgM, and IgG were significantly increased in the treatment groups compared to the control (*P* < 0.05). The highest values for IL-10 and IgM were observed in the 8% and 12% FSPR groups, while the highest IgG level was found in the 8% FSPR group. Furthermore, these three parameters (IL-10, IgM, and IgG) exhibited both significant linear and quadratic increasing trends (*P* < 0.05). Regarding metabolic parameters, no significant differences were observed in liver function (ALT, AST), muscle enzymes (CK), glucose (Glu), or triglycerides (TG) among the treatments (*P* > 0.05). However, for Total Cholesterol (TC), although the ANOVA revealed no significant difference among means (*P* = 0.175), a significant linear decreasing trend was observed with increasing FSPR inclusion levels (*P* = 0.023).

**Table 8:** Effects of dietary FSPR on serum biochemical parameters in geese.

Items	CON	5%FSPR	8%FSPR	12%FSPR	ANOVA (P)	Linear (P)	Quadratic (P)
**Immunoglobulins**							
IL-10, pg/mL	5.48 ± 0.35^b^	5.52 ± 0.36^b^	6.76 ± 0.34^a^	6.92 ± 0.43^a^	0.016	0.004	0.018
IgM, μg/mL	169.33 ± 11.08^c^	187.44 ± 11.50^b^	221.30 ± 13.98^a^	208.05 ± 12.86^a^	0.038	0.016	0.035
IgG, μg/mL	138.32 ± 10.35^c^	148.15 ± 6.93^b^	177.75 ± 11.72^a^	162.34 ± 5.56^b^	0.030	0.030	0.056
**Liver Function**							
ALT, U/L	19.95 ± 2.78	23.60 ± 6.76	18.40 ± 2.25	25.65 ± 10.34	0.843	0.631	0.851
AST, U/L	21.8 ± 0.28	21.66 ± 0.82	21.18 ± 1.06	21.82 ± 0.21	0.905	0.894	0.862
**Muscle Enzymes**							
CK, U/L	1348.02 ± 261.08	1521.7 ± 287.98	1114.83 ± 280.73	2115.28 ± 293.58	0.099	0.154	0.126
**Lipid Profile**							
TC, mmol/L	4.98 ± 0.21	4.81 ± 0.19	4.61 ± 0.15	4.44 ± 0.15	0.175	0.023	0.081
TG, mmol/L	0.6 ± 0.020	0.55 ± 0.02	0.53 ± 0.02	0.55 ± 0.02	0.258	0.139	0.128
**Glucose Metabolism**							
Glu, mmol/L	10.79 ± 0.36	11.41 ± 0.73	9.40 ± 0.58	10.29 ± 0.50	0.102	0.258	0.528

Data are presented as mean ± SEM (n = 6). Means within a row with different lowercase superscripts differ significantly (*P* < 0.05).

Note: Other measured parameters, including pro-inflammatory cytokines (IL-1β, IL-6, IL-8, TNF-α), liver enzymes (ALT, AST), and muscle enzymes (CK), showed no significant differences among treatments (P > 0.05) and are not presented here for brevity.

#### Intestinal morphology

**Fig. 1:**
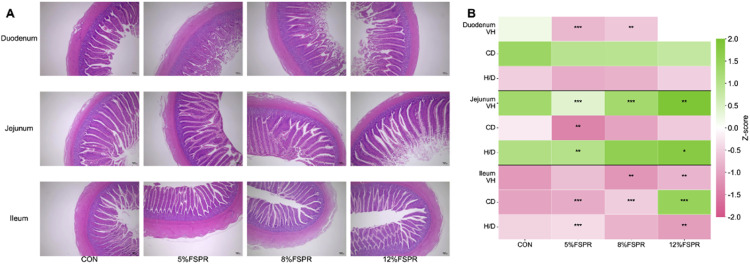
Effects of dietary FSPR on intestinal morphology of geese.

The effects of dietary FSPR on intestinal morphology are summarized in Table 9 and visually represented in Fig. 1.

**Table 9:** Effects of dietary FSPR on intestinal morphology of geese.

Items	CON	5%FSPR	8%FSPR	12%FSPR	ANOVA (P)	Linear (P)	Quadratic (P)
**Duodenum**							
VH, μm	148.19 ± 43.23^a^	122.25 ± 33.94^b^	128.06 ± 32.97^b^	155.22 ± 51.03^a^	< 0.001	0.843	0.041
CD, μm	36.89 ± 9.10	37.56 ± 8.25	37.47 ± 9.52	39.34 ± 9.44	0.486	0.111	0.285
H/D	4.13 ± 1.26	3.62 ± 1.98	3.69 ± 1.48	4.09 ± 1.58	0.177	0.902	0.058
**Jejunum**							
VH, μm	212.12 ± 31.84^a^	160.95 ± 31.39^c^	190.81 ± 28.0^b^	191.4 ± 49.48^b^	< 0.001	0.710	0.615
CD, μm	31.23 ± 6.10^a^	27.78 ± 6.09^b^	29.38 ± 7.00^b^	32.55 ± 6.50^a^	< 0.001	0.727	0.183
H/D	7.06 ± 1.76^a^	6.09 ± 1.90^b^	6.82 ± 1.91^a^	6.17 ± 2.06^b^	0.011	0.405	0.778
**Ileum**							
VH, μm	121.28 ± 20.67^a^	125.06 ± 34.39^a^	109.14 ± 19.54^b^	111.14 ± 14.80^b^	< 0.001	0.281	0.678
CD, μm	39.73 ± 10.62^a^	29.78 ± 6.64^b^	31.25 ± 8.02^b^	29.33 ± 8.53^b^	< 0.001	0.159	0.304
H/D	3.36 ± 1.40^c^	4.26 ± 1.06^a^	3.70 ± 1.07^b^	4.17 ± 1.43^a^	< 0.001	0.356	0.702

Data are presented as mean ± SEM (n = 6). Means within a row with different lowercase superscripts differ significantly (*P* < 0.05).

Abbreviations: VH, villus height; CD, crypt depth; H/D, villus height to crypt depth ratio.

(A) Representative photomicrographs of small intestinal villi (hematoxylin and eosin (H&E) staining; scale bar = 100 μm). (B) Heatmap of villus height (VH), crypt depth (CD), and the ratio of VH to CD (H/D) across duodenal, jejunal, and ileal segments. Data are presented as Z-score normalized values.

In the duodenum, the VH was significantly affected by the treatment (*P* < 0.001). Specifically, the VH was lower in the 5% and 8% FSPR groups compared to the CON group, but recovered to a level comparable to the CON group at the 12% inclusion level. This fluctuation followed a significant quadratic trend (*P* = 0.041). No significant differences were observed in CD or the villus height to H/D in this segment (*P* > 0.05).

In the jejunum, significant differences were found in all parameters (*P* < 0.05). The CON group exhibited the highest VH, whereas the FSPR groups showed reduced values. However, regarding CD, the 5% and 8% FSPR groups showed significantly lower values compared to the CON and 12% FSPR groups.

In the ileum, significant treatment effects were observed for VH, CD, and H/D (P < 0.001). VH was significantly lower in the 8% and 12% FSPR groups compared to CON and 5% FSPR. CD was significantly greater in the CON group than in all FSPR-supplemented groups. Conversely, the H/D ratio was significantly higher in the 5% and 12% FSPR groups compared to CON and 8% FSPR.

The most notable morphological alterations were observed in the ileum, as clearly reflected in the heatmap ( Fig. 1B). VH was lower in the 8% and 12% FSPR groups compared to the CON and 5% FSPR groups (*P* < 0.05). Conversely, CD was markedly reduced across all FSPR-supplemented groups compared to the CON group (*P* < 0.05). This divergent change resulted in a significantly increased H/D ratio in all FSPR-supplemented groups (*P* < 0.05), wherein the 5% and 12% FSPR groups achieved higher ratios than the 8% FSPR group. These structural alterations are consistent with the villus morphology observed in the histological sections ( Fig. 1A).

#### Taxonomic composition of cecal microbial communities

**Fig. 2:**
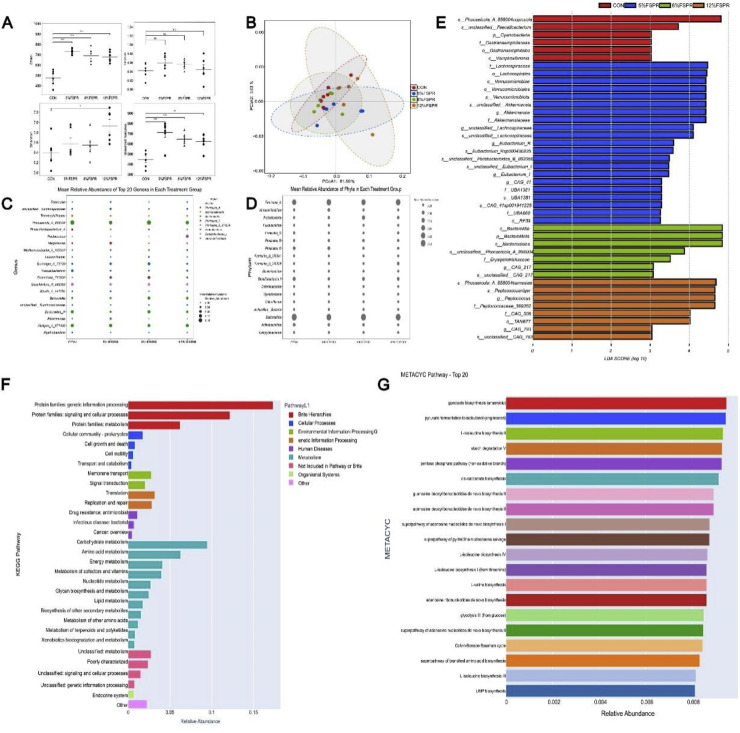
Effects of dietary FSPR on the cecal microbiota of geese.

Dietary supplementation with FSPR substantially reshaped the composition, structure, and metabolic function of the cecal microbiota in geese, as comprehensively detailed in Fig. 2. The alpha diversity of the microbiota was significantly enhanced by FSPR ( Fig. 2A). The Observed features and Chao1 indices were significantly higher in all FSPR groups compared to the CON group (*P* < 0.001 and *P* < 0.01, respectively). The 12% FSPR group exhibited a significantly greater Shannon index compared to the CON, 5%, and 8% FSPR groups (*P* < 0.05). In contrast, no significant differences were found for the Simpson index.

(A) Alpha diversity indices, including the Chao1, Shannon, Simpson, and Observed features indices. Data are presented as mean ± SEM (n = 6). (B) Principal coordinates analysis (PCoA) based on weighted UniFrac distance. The percentage of variance explained by each coordinate is indicated on the axes. (C) Relative abundance of the top 20 genera at the genus level. (D) Relative abundance of bacterial communities at the phylum level. (E) Histogram of linear discriminant analysis (LDA) effect size (LEfSe) scores for differentially abundant taxa (LDA score > 2.0). (F) Relative abundance of predicted Kyoto Encyclopedia of Genes and Genomes (KEGG) metabolic pathways at pathway level 2. (G) Relative abundance of the top 20 predicted MetaCyc pathways ranked by abundance. *P < 0.05, **P < 0.01, ***P < 0.001; ns, not significant.

The overall microbial community structure was significantly reshaped, as demonstrated by principal coordinates analysis (PCoA), with clear separation between treatments (analysis of similarity, ANOSIM, *P* = 0.001; Fig. 2B). Substantial shifts in microbial composition were observed at both the phylum and genus levels ( Fig. 2C, D). At the phylum level, FSPR treatment increased the relative abundance of *Bacteroidota* while decreasing that of *Proteobacteria*. At the genus level, FSPR supplementation markedly increased the abundance of beneficial genera, including *Phocaeicola* and unclassified members of the family *Lachnospiraceae*. The linear discriminant analysis effect size (LEfSe) analysis confirmed these findings, identifying multiple bacterial taxa as statistically significant biomarkers (LDA score > 2.0) for the FSPR-treated groups, primarily featuring species from the genus *Phocaeicola* and members of the family *Lachnospiraceae* (linear discriminant analysis (LDA) score > 2.0) for the FSPR-treated groups ( Fig. 2E).

Predictive functional analysis revealed that these compositional changes were directly linked to significant metabolic alterations. Kyoto Encyclopedia of Genes and Genomes (KEGG) pathway analysis showed an increase in the abundance of pathways related to "Carbohydrate metabolism" and "Amino acid metabolism" ( Fig. 2F). A more detailed MetaCyc pathway analysis indicated the upregulation of key pathways for "starch degradation V" and the biosynthesis of branched-chain amino acids (BCAAs), such as the “superpathway of branched amino acid biosynthesis” ( Fig. 2G).

Taken together, these results demonstrate that FSPR inclusion enriches for beneficial bacterial taxa and enhances microbial metabolic pathways involved in carbohydrate utilization and amino acid synthesis.

## Discussion

In the present study, the inclusion of FSPR linearly decreased ADFI while improving FCR, with the 8% inclusion level achieving optimal body weight gain. This “eat less, grow more” phenomenon suggests a significant improvement in nutrient utilization efficiency. The reduction in feed intake may be attributed to the high dietary fiber content in FSPR, which physically promotes satiety (Do et al., 2023) or potentially due to the fermentation-derived organic acids regulating appetite (Kang et al., 2022). However, unlike conventional high-fiber diets that often suppress growth, FSPR supplementation maintained ADG. From a morphological perspective, this resilience may be supported by our intestinal findings (Table 9). Although duodenal villus height was compromised, the ileum exhibited a compensatory improvement in absorption capacity, as evidenced by the significantly increased H/D ratio. The ileum is a critical site for the absorption of fermentation end-products and recirculated nutrients(Mantle, 2020). Therefore, the improved micro-structure in the hindgut likely compensated for the reduced intake, maximizing nutrient extraction efficiency.

Beyond enhancing nutrient utilization, the improvements in intestinal health and nutrient absorption capacity were also reflected in the quality of meat. Meat pH and color are pivotal indicators of physiological homeostasis during the conversion of muscle to meat. We observed that FSPR, particularly at 12%, significantly maintained a higher pH in thigh muscle at 24 h postmortem. A rapid pH decline is typically associated with accelerated anaerobic glycolysis and protein denaturation, leading to pale, soft, and exudative (PSE) meat. The higher pH and improved color stability (lower L* values) in FSPR groups suggest a delay in postmortem glycogen degradation (Rathgeber et al., 1999). This protective effect may be partly attributed to the potential antioxidant properties of fermented feed. Fermentation releases bound phenolics and generates microbial metabolites that can mitigate oxidative stress (Akbari et al., 2023). Although we did not measure tissue antioxidant markers directly, the systemic reduction in serum cholesterol (Table 8) and the stable levels of liver enzymes indicate a healthier metabolic status. We speculate that this improved metabolic state may support muscle cell membrane integrity and reduce drip loss, thereby preserving meat quality(Zhu et al., 2020).

The observed systemic metabolic benefits and antioxidant potential prompted us to investigate whether FSPR influenced the host's immune status, which is often closely tied to gut health and metabolic inflammation. A major concern with using agricultural by-products is the potential induction of systemic inflammation due to anti-nutritional factors (Samtiya et al., 2020). Strikingly, our serum biochemical analysis (Table 8) revealed that FSPR acted as an immune booster rather than a stressor. The dose-dependent increase in serum immunoglobulins (IgM, IgG) and the anti-inflammatory cytokine IL-10 demonstrates a robust activation of humoral immunity (Guthmiller et al., 2017). This outcome appears to be associated with gut microbiota remodeling observed in our study. The proliferation of *Lachnospiracea*e (Fig. 2E) is biologically significant, as this family is a renowned producer of butyrate, a potent anti-inflammatory mediator that regulates regulatory T cells (Tregs) and IL-10 production(Sarıtaş et al., 2024). Therefore, these results suggest that FSPR may act as an immunomodulatory additive, enhancing humoral and anti-inflammatory responses while not inducing a sustained pro-inflammatory state, as evidenced by the unaltered levels of IL-1β and TNF-α.

Collectively, our findings present a coherent picture in which dietary inclusion of FSPR reshapes the gut ecosystem. This change triggers a cascade of beneficial effects, ranging from compensatory ileal adaptation that improves nutrient extraction to systemic antioxidant and anti-inflammatory states that enhance meat quality and immune competence. Ultimately, these effects explain the paradox of reduced intake yet sustained growth. A limitation of this study is the lack of direct nutrient digestibility analysis and cecal SCFA quantification. Future studies will address these gaps to confirm the proposed metabolic mechanisms.

## Conclusion

In summary, dietary supplementation with FSPR improved feed efficiency by triggering compensatory ileal absorption to offset reduced intake, enhanced meat quality attributes through metabolic and antioxidant regulation, and modulated the gut microbiota to boost systemic immunity without inflammatory costs. Based on the present findings, considering the optimal growth performance and nutrient utilization, a dietary inclusion level of 8% FSPR is suggested for practical application. Nevertheless, further studies are warranted to directly quantify tissue antioxidant markers and elucidate the exact mechanism by which fermentation metabolites regulate satiety signals. Overall, this study supports the potential of FSPR as a sustainable and functional feed strategy, contributing to the valorization of agricultural by-products and the development of healthy, antibiotic-free animal production systems.

## CRediT authorship contribution statement

**Jiayi Xu:** Writing – review & editing, Writing – original draft, Visualization, Validation, Supervision. **Chao Wang:** Data curation. **Guoan Yin:** Methodology. **Shuai Zhao:** Project administration. **Xin Liu:** Software. **Weiguo Cui:** Conceptualization.

## Disclosures

All authors disclosed no relevant relationships.
